# Detailed characterization of Redondovirus in saliva of SARS-CoV-2-infected individuals in Sao Paulo, Brazil

**DOI:** 10.1371/journal.pone.0291027

**Published:** 2023-08-31

**Authors:** Antonio Charlys da Costa, Maria C. Mendes-Correa, Tania Regina Tozetto-Mendoza, Lucy S. Villas-Boas, Anderson Vicente de Paula, Heuder Gustavo Oliveira Paiao, Fabio E. Leal, Noely E. Ferreira, Layla Honorato, Elcio Leal, Giuliano Grandi, Vanessa dos Santos Morais, Erika R. Manuli, Ester C. Sabino, Steven S. Witkin

**Affiliations:** 1 Laboratório de Investigação Médica em Virologia, Instituto de Medicina Tropical, Faculdade de Medicina da Universidade de São Paulo, São Paulo, Brazil; 2 Departamento de Molestias Infecciosas e Parasitarias da Faculdade de Medicina da Universidade de São Paulo, São Paulo, Brazil; 3 Faculdade de Medicina da Universidade Municipal de Sao Caetano do Sul, São Paulo, Brazil; 4 Programa de Oncovirologia, Instituto Nacional de Câncer, Rio de Janeiro, Brazil; 5 Laboratório de Diversidade Viral, Instituto de Ciências Biológicas, Universidade Federal do Pará, Pará, Brazil; 6 Universidade Federal de São Paulo, São Paulo, Brazil; 7 Department of Obstetrics and Gynecology, Weill Cornell Medicine, New York, NY, United States of America; Shanghai Public Health Clinical Center, Fudan University, CHINA

## Abstract

**Background:**

Redondovirus (ReDoV) is a DNA virus present in the respiratory tract of many healthy individuals. Since SARS-CoV-2, the virus responsible for COVID-19, also primarily infects the same site, we evaluated whether ReDoV was present at increased frequency in patients with COVID-19 and influenced infection parameters.

**Methods:**

Saliva samples were collected weekly from 59 individuals with COVID-19 and from 132 controls. ReDoV was detected by polymerase chain reaction and the genotypes were identified by metagenomics. Torque Teno Virus (TTV) in these samples were previously reported.

**Results:**

ReDoV was detected in saliva more frequently from COVID-19 patients (72.9%) than from controls (50.0%) (p = 0.0015). There were no associations between ReDoV detection and either continuous or intermittent SARS-CoV-2 shedding, the duration of SARS-CoV-2 detection in saliva, patients’ sex or if infection was by the B1 or Gamma strain. The two ReDoV strains, Brisavirus and Vientovirus, were present in equivalent frequencies in ReDoV-positive COVID-19 patients and controls. Phylogenetic analysis suggested that the two ReDoV strains in Brazil were similar to strains previously detected on other continents.

**Conclusion:**

ReDoV expression in saliva is increased in males and females in Brazil with mild COVID-19 but its presence does not appear to influence properties of the SARS-CoV-2 infection.

## Introduction

Redondovirus (ReDoV), a member of the *Redondoviridae* family in the phylum Cressdnaviricota, is a circular, Rep-encoding single-stranded (CRESS) small DNA virus. It is currently composed of two species, Brisavirus and Vientovirus, and is present primarily in the respiratory tract of healthy individuals [1, 2]. It has also been reported to occur at increased concentrations in some individuals with immunological alterations, periodontal disease or serious illnesses [2–4]. However, its apparent lack of consistent association with adverse conditions has led to the designation of ReDoV as a non-pathogenic commensal human virus [1, 2]. It remains uncertain, similar to other viral co-infections [5], whether the presence of ReDoV can influence, in either a positive or negative manner, the activity and pathogenic potential of other concurrent viral infections. It has been shown that the replication of respiratory syncytial virus is influenced by the extent of respiratory tract inflammation [6].

Severe acute respiratory syndrome coronavirus-2 (SARS-CoV-2) is the virus responsible for the coronavirus disease -2019 (COVID-19) pandemic. Its initial and primary site of infection is the human respiratory tract [7]. Disease severity, length of illness and duration of viral shedding and infectivity varies greatly between infected individuals [8]. Several studies have evaluated whether the presence of Torque Teno Virus (TTV), another endogenous DNA virus residing in the respiratory tract of many individuals, influences the course of SARS-CoV-2 infection [9–13]. The results to date have been mostly negative. The potential influence of concurrent ReDoV colonization on properties of a SARS-CoV-2 infection has received less attention. A study of saliva from Italy [13] reported that ReDoV was identified in 61% of subjects, and was more prevalent in a control group (65%) than in COVID-19 patients (52%). A study from the United States [4] utilizing oropharyngeal, nasopharyngeal and endotracheal samples determined that levels of ReDoV increased with disease severity in COVID-19 patients and were highest in those patients who were intubated. In neither study were the ReDoV species identified nor was a phylogenetic analysis performed.

The present study evaluated the presence in saliva of ReDoV and its two species, Brisavirus and Vientovirus, and its influence on properties of a COVID-19 infection in outpatients in Brazil. A phylogenetic analysis of the two ReDoV species detected was also performed.

## Materials and methods

### Subjects

In this observational study the convenience samples were obtained from individuals who were participating in a primary care initiative, *The Corona São Caetano Program*, offering COVID-19 testing and care to residents of São Caetano do Sul, Brazil [14]. Details of the study population were previously described [9]. Briefly, inclusion criteria for subjects and controls included the detection or absence, respectively, of SARS-CoV-2 on a nasopharyngeal swab by polymerase chain reaction, aged >18 years, the absence of any immunosuppressive, endocrine or systemic infectious disorder, malignancy or pregnancy and the ability to provide informed written consent. Individuals with any of the above parameters were excluded. The authors did not have access to the information that could identify individual participants during or after data collection.

### Ethical approval

The study was approved by the local ethics committee: Comissão Nacional de É tica em Pes- quisa do Ministário da Saúde do Brasil (CONEP), protocol No. CAAE 30419320.7.0000.0068, dated April, 18, 2020 and all subjects provided written informed consent.

### Saliva collection

Saliva samples were collected using a cotton pad device–Salivette™ (Sarstedt AG & CO. KG, Nümbrecht, Germany), as reported previously [9]. Briefly, the participants were instructed to vigorously chew a cotton pad for one minute before its placement into a separate tube. The tubes with cotton samples were centrifuged at 800 g and the salivary fluid extracted from the cotton was subjected to nucleic acids extraction and purification. The samples were collected during the morning hours and participants were instructed to avoid eating, drinking or brushing their teeth at least one hour before the saliva collection. Samples were immediately put in a cool box (2–8°C) and stored at 4°C in a refrigerator until shipment to the lab by a specialized carrier in the afternoon the same day.

### RNA extraction, PCR amplification

All samples were handled according to laboratory biosafety guidelines. Saliva samples were subjected to total nucleic acid extraction with the QIAamp viral RNA kit (QIAGEN, Hilden, Germany), according to the manufacturer’s instructions. All samples were suitable for amplification, as assessed by successful amplification of an internal control. Samples were then subjected to RT-PCR (RealStar® SARS-CoV-2 RT-PCR Kit 1.0, Altona Diagnostics*)* followed by thermocycling using the Roche LightCycler® 96 System.

### ReDoV and TTV detection

For ReDoV detection in saliva, a specific probe and primers were utilized, as previously described [3]. Briefly, a PCR assay was designed targeting the genomic region encoding the capsid gene (ReDoVF GCAGAGTTGTCAGCACATTT; ReDoVR, ATACCAGTATAGGAAGATTTCGAG; ReDoVProbe FAM-AAATGGAAGGGAGAGAGGCCTTTGG-BHQ). For each sample, 20 μL reactions containing 5μL of template DNA, 0.33μL forward and reverse primers (18μM), 0.33μL probe (5μM), 10μL TaqMan Fast Universal PCR Master Mix (Thermo Fisher Scientific, Warrington, UK) and 5 μL water were analyzed on a QuantStudio 5 Real Time PCR System (Thermo Fisher Scientific, Warrington, UK) with the following cycling profile: 20 sec at 95°C for 1 cycle, and 40 cycles of 95°C for 3 sec and 60°C for 30 sec (signal collection). The ReDoV DNA amplification was based on the TaqManTM Universal PCR master mix protocol (Thermo Fisher Scientific, Warrington, UK). The data were analyzed using QuantStudio Design & Analysis Software v.1.4.1. Positive and negative controls for ReDoV in saliva were obtained from samples stored in the repository at the Virology Laboratory (Institute of the Tropical Medicine of the Medicine School of the São Paulo University, Brazil

The detection of TTV in these saliva samples has been reported previously [9]. TTV-specific DNA was amplified using as target a segment of the untranslated region (UTR) of the viral genome using primers and a probe as previously described by Maggi et al. [15]: forward primer 5′-GTGCCGIAGGTGAGTTTA-3′; reverse primer 5′-AGCCCGG CCAGTCC-3′; probe 5′-FAM TCAAGGGGCAATTCGGGCT-MGBNFG 3′. The real time quantitative PCR (qPCR) was performed with 0.25 μM of each primer, 0.062 μM of probe and ~100 ng de DNA template in final volume of 25 μL in TaqMan^®^ Universal Master mix (Thermo Fisher Scientific, Warrington, United Kingdom), according to the manufacturer’s instructions. Thermocycling conditions consisted of two initial heat activation steps of 50°C for 2 min and 95°C for 15 s, followed by 50 cycles of 15 s at 95°C and 1 min at 60°C, in a Quantstudio^™^ 5 instrument. For TTV quantification, the TTV qPCR standard curve was generated using known amounts of HPLC purified synthetic DNA of 73 bp, commercially synthesized (Exxtend Biotecnology Ltda, São Paulo, Brazil): 5′-TTCGTAGCCCGGCCAGTCCCGTATAGCCCGAATTGCCCCTTGAATGCGTTAAACTCACC AI CGGCACCTGATA-3′. Data were analyzed using QuantStudio Design & Analysis Software v.1.4.1.The lower limit of detection of the qPCR assay for TTV was 40 copies per ml (1.6 log_10_ copies/ml).

### SARS-CoV-2 strain detection

SARS-CoV-2 strain detection employed a customized SARS-CoV-2 mutation panel by using TaqMan SNP Genotyping Assays with TaqPath one-step RT-PCR reaction (Applied Biosystems^TM^) and was performed, according to manufacturer’s instructions. The data were analyzed by QuantStudio Design and Analysis Software v2.5 with the Genotyping Analysis Module. SARS-CoV-2 complementary DNA was also sequenced using the MinION platform (Oxford Nanopore Technologies, UK) and Miseq (Illumina, San Diego, CA, USA) to detect the B1 and Gamma strain variants [16]. The data was analyzed by QuantStudio Design and Analysis Software v2.5 with the Genotyping Analysis Module.

### ReDoV genotype characterization and phylogenetic analysis

Samples were subjected to circular nucleic acid enrichment using the TempliPhi™ Kit (Cytiva, Little Chalfont, London, England), following the manufacturer’s instructions. The samples were then quantified by fluorimetry, subjected to library preparation with the Nextera XT Kit (Illumina, San Diego, CA, USA) and subsequently sequenced with the MiSeq Reagent Kit v3 (Illumina, San Diego, CA, USA), following the manufacturer’s instructions.

A pipeline was used to analyze sequence data. Before analyzin’, raw data were pre-processed by subtracting human and bacterial sequences, duplicate sequences, and low quality reads. Following de novo assembly using the Ensemble program [17] both contigs and singlets, viral sequences were then analyzed using translated protein sequence similarity search (BLASTx v.2.2.7) to all annotated viral proteins available in GenBank. Candidate viral hits were then compared to an in-house non-virus non-redundant protein database to remove false positive viral hits. The Geneious R9 program was used to align reads and contigs to reference viral genomes from GenBank and generate complete or partial genome sequences.

Phylogenetic trees were constructed using the maximum likelihood approach, and branching support was estimated using a bootstrap test with 1000 replications using the IQ-Tree tool [https://doi.org/10.1093/nar/gkw256.]. Trees were visualized and edited using Figtree version 1.4.2 [18].

### Statistics

Differences in discrete variables between individuals positive or negative for RedoV and SARS-CoV-2 were analyzed by Fisher’s exact test. Continuous variables were analyzed by the Mann-Whitney test. Differences in the ReDoV level over time was analyzed by the Kruskal-Wallis test. A p value <0.05 was considered significant. The GraphPad Prism 9 software (San Diego, CA) was used for all analyses.

## Results

The rate of detection of ReDoV in saliva from COVID-19 patients and controls are described in Table 1. ReDoV was identified in 74.6% of individuals with COVID-19 as opposed to 50.0% of controls (p = 0.0015). There was no association between subjects’ gender and COVID-19 and ReDoV status, i.e., 76.7% of males and 71.4% of females were positive for ReDoV. The mean age of all subjects was between 39.7 and 42.8 years and did not differ between subjects in both patients and controls who were positive or negative for ReDoV. The percentage of subjects poaitive for ReDoV were higher in both male and fenale COVID-19 patients than in controls, but this only reached statistical significance in males (p = 0.0486).

**Table 1:** Characteristics of COVID-19 patients and controls positive or negative for Redondovirus.

Characteristic	COVID-19	Controls	p value
	N = 59	N = 132	
Redondovirus positive	44 (74.6%)	66 (50.0%)	0.0015
Mean age (SD)			
Redondovirus positive	40.5 (12.5)	40.6 (13.3)	NS
Redondovirus negative	41.1 (19.7)	42.3 (16.9)	NS
Redondovirus positive males	13/17 (76.7%)	23/49 (46.9%)	0.0486
Redondovirus positive females	30/42 (71.4%)	43/83 (51.8%)	0.0540

NS, not significant

Characteristics of variables associated with subjects’ SARS-CoV-2 infection in COVID-19 patients who were positive or negative for ReDoV are given in Table 2. There was no association between the presence of ReDoV and the shedding pattern of SARS-CoV-2. Of the 40 individuals whose shedding of SARS-CoV-2 was consistent over a 4–5 week period, 75% were ReDoV positive. Similarly, in the 19 individuals with only intermittent SARS-CoV-2 shedding 68.4% were ReDoV positive (p = 0.999). The B1 strain of SARS-CoV-2 was responsible for the infection in 34 individuals, while 25 were infected with the SARS-CoV-2 Gamma strain. No differences were detected in the percent of subjects positive for ReDoV between the two SARS-CoV-2 strains (p = 0.1408). The length of time that SARS-CoV-2 was detected in saliva samples varied among the subjects from 2 to >40 days and no associations were detected between the presence of ReDoV and the duration of shedding.

**Table 2:** Characteristics of COVID-19 patients positive for Redondovirus.

Characteristic	No. ReDoV positive/No. detected (%)
Continuous SARS shedding	30/40 (75.0%)
Intermittent SARS shedding	13/19 (68.4%)
B1 strain infection	22/34 (64.7%)
Gamma strain infection	21/25 (84.0%)
Duration SARS positive (days)	
2–10	15/18 (83.3%)
11–20	10/15 (66.7%)
21–30	9/13 (69.2%)
31–40	7/9 (77.8%)
>40	2/4 (50.0%)

Metagenomic analysis for the determination of ReDoV species was performed on 28 saliva samples from COVID-19 patients and on 29 control samples that were positive for ReDoV by PCR. Brisavirus was identified in 67.9% of patients and 72.4% of controls, while Vientovirus was present in 64.3% of patients and 79.3% of controls (Table 3). None of these differences were significant. TTV was present in 75.9% of controls and 57.1% of COVID-19 patients who were positive for ReDoV. The difference was not significant (p = 01685).

**Table 3:** Redondovirus species in saliva from individuals with COVID-19 and controls.

Species detected	Controls	COVID-19
	N = 29	N = 28
Brisnavirus + Vientovirus	15 (51.7%)	9 (32.1%)
Brisnavirus only	6 (20.7%)	10 (35.7%)
Vientovirus only	8 (27.6%)	9 (32.1%)
Brisnavirus total	21 (72.4%)	19 (67.9%)
Vientovirus total	23 (79.3%)	18 (64.3%)

The presence of the two species of redondovirus were determined by metagenomic analysis.

TTV was identified in 62.5% and 72.7% of patients and controls, respectively, that were positive for Brisavirus, and 81.3% and 77.3% of patients and controls, respectively, positive for Vientovirus (Table 4).

**Table 4:** Relationship between Redondovirus species in saliva and salivary Torque Teno Virus.

Species detected	Controls		COVID-19	
	TTV+	TTV-	TTV+	TTV-
	N = 22	N = 7	N = 16	N = 12
Brisavirus + Vientovirus	11 (50.0%)	4 (57.1%)	7 (43.8%)	2 (16.7%)
Brisavirus only	5 (22.7%)	1 (14.3%)	3 (18.8%)	7 (58.3%)
Vientovirus only	6 (27.3%)	2 (28.6%)	6 (37.5%)	3 (25.0%)
Brisavirus total	16 (72.7%)	5 (71.4%)	10 (62.5%)	9 (75.0%)
Vientovirus total	17 (77.3%)	6 (85.7%)	13 (81.3%)	5 (41.7%)

TTV, torquetenovirus TTV was detected by qPCR while Brisavirus and Vientovirus were detected by metagenomics.

There were no differences in the rates of Brisavirus or Vientovirus detection between patients and controls by sex (S1 Table) or by age (S2 Table).

**Fig 1:**
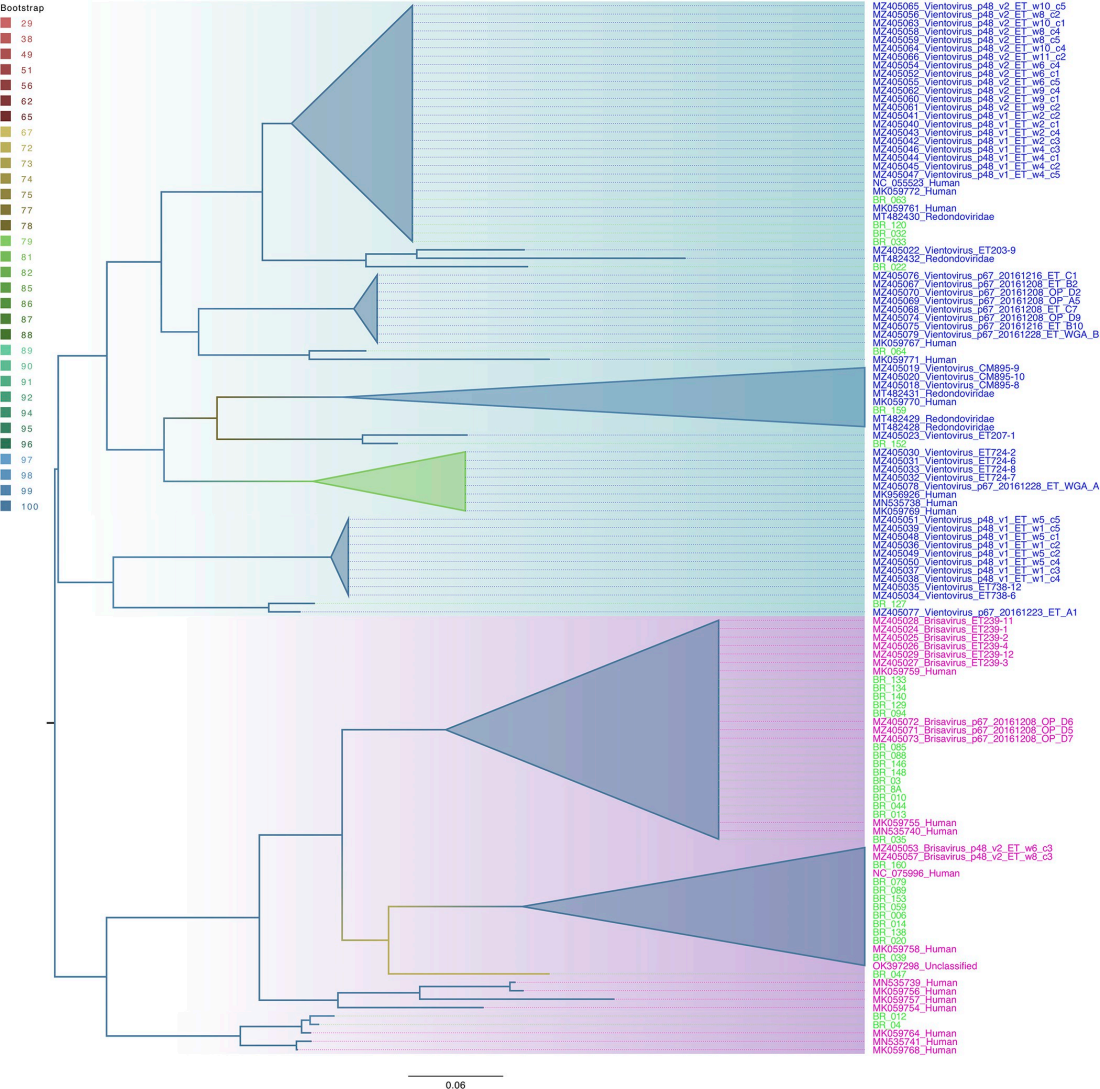
Phylogenetic tree of redondoviruses. The tree was constructed based on full-length genomes, utilizing the maximum likelihood approach. The evolutionary model employed for the analysis was the GTR model with gamma correction. Branches in the tree were color-coded according to the bootstrap support, as indicated on the scale. Sequences associated with Vientovirus are highlighted in blue, while sequences associated with Brisavirus are highlighted in magenta. Sequences generated in this study were highlighted in green. Horizontal bar indicates the scale of the tree in nucleotide substitutions per site.

Phylogenetic analysis revealed that twenty six of the ReDoV sequences analyzed in this study showed a close relationship with Brisavirus (Fig 1, represented by the magenta color in the tree). Additionally, ten other sequences were found to be related to Vientovirus (Fig 1, represented by the blue color in the tree). It is important to note that Brisavirus and Vientovirus are two distinct species belonging to the genus Torbevirus, despite sharing a nucleotide similarity of 50%.

## Discussion

ReDoV was detected in saliva from 74.6% of individuals infected with SARS-CoV-2, a significantly greater frequency than found in the control population. This observation is consistent with the hypothesis that conditions permissive for SARS-CoV-2 infection, such as localized inflammation and/or alterations in mucosal integrity, may simultaneously also promote ReDoV replication and/or persistence. Further testing is necessary to verify or refute this possibility. Among COVID-19 patients we could not detect any differences in the pattern of SARS-CoV-2 expression between those positive or negative for ReDoV. This indicates that, limited to the analyses that we performed, ReDoV did not appear to influence properties of SARS-CoV-2 that were present in saliva. In contrast to our observations, a study from Italy reported that ReDoV was detected more frequently in saliva from controls than in those with COVID-19 [13]. A United States-based study using swab samples from several respiratory tract sites found that the occurrence of ReDoV in COVID-19 patients increased with disease severity [4]. Differences in results between these studies may have been due to several factors including patient selection, geographical differences in rates of infection, overall health status, genetic variations, assay sensitivity and methods of sample acquisition and handling. Nevertheless, what appears to be consistent is that there is no evidence that the presence of ReDoV influences properties of SARS-CoV-2 infection or the course of COVID-19.

In our Brazilian subjects, ReDoV was detected in saliva in 50.0% of those who were negative for the SARS-CoV-2 virus. It has been reported that 32% of saliva samples from healthy individuals in the United States, and 70% of saliva samples from individuals from countries in Africa were ReDoV positive. The same authors reported that Vientovirus was the predominant ReDoV species detected in samples from the United States and Europe, while approximately equal frequencies of Brisavirus and Vientovirus were detected in individuals from Ethiopia and China [19]. Combined with our findings, these observations suggest that there may be geographical differences in ReDoV species prevalence in the nasopharynx. Also, our observations are consistent with previous reports that both Brisavirus and Vientovirus are equally prevalent, multiple individuals are colonized by both strains simultaneously and that they persist over time [19]. It has been suggested that the persistence of the two independent strains of ReDoV indicates that both most likely are long time inhabitants in the human respiratory tract [19].

By phylogenetic analysis, we did not observe a direct relationship between ReDoV species in the study samples. This suggests that the species present are members of closely related groups, similar to the homogeneous distribution between ReDoV species previously described in other continents (Asia, Africa, China and Europe) [19]. This strongly indicates that the species identified in the present study were descended from ancestors that were probably introduced into Brazil long before the first description of these viral species in 2017–2018.

A major limitation of our investigation is the small sample size and that all COVID-19 subjects had mild disease that did not require hospitalization. Comparable measurements in saliva on subjects with severe COVID-19, while reported in other geographic regions [19], have yet to be determined in Brazil. In addition, we did not evaluate our subjects for periodontal disease, a condition reported to be associated with elevated ReDoV levels [2]. Although we know of no evidence linking COVID-19 with periodontitis, this possible association remains to be evaluated. Lastly, the viral load of ReDoV was not measured in these subjects, so we were unable to determine if there was a possible correlation between ReDoV viral load and disease severity in the COVID-19 patients.

In conclusion, ReDoV and its two species, while more prevalent in individuals in Brazil with mild COVID-19 than in controls, had no detectable influence on properties of the SARS-CoV-2 infection.

## Supporting information

S1 Checklist (DOCX)

S1 Table Redondovirus species in saliva from male and female COVID-19 patients and controls positive for Redondovirus. (DOCX)

S2 Table Association between Redondovirus species in saliva and age in individuals with COVID-19 and controls who were positive for Redondovirus. (DOCX)
