# Supplementary material for: Detailed characterization of Redondovirus in saliva of SARS-CoV-2-infected individuals in Sao Paulo, Brazil
Source: PLoS One. 2023 Aug 31;18(8):e0291027. doi: 10.1371/journal.pone.0291027 (PMC10470920; doi:10.1371/journal.pone.0291027)
Supplement: S1 Table — (DOCX) [file pone.0291027.s002.docx]

S1 Table. Redondovirus species in saliva from male and female COVID-19 patients and controls positive for Redondovirus

Species detected Controls COVID-19

Females Males Females Males

N = 22 N = 7 N =20 N =7

Brisavirus + Vientovirus 12 (54.5%) 3 (42.9%) 6 (30.0%) 3 (42.9%)

Brisavirus only 4 (18.2%) 2 (28.6%) 9 (45.0%) 1 (14.3%)

Vientovirus only 6 (27.3%) 2 (28.6%) 5 (25.0%) 3 (42.9%)

Brisavirus total 16 (72.7%) 5 (71.4%) 15 (75.0%) 4 (57.1%)

Vientovirus total 18 (81.8%) 5 (71.4%) 11 (55.0%) 6 (85.7%)
