# Supplementary material for: Detailed characterization of Redondovirus in saliva of SARS-CoV-2-infected individuals in Sao Paulo, Brazil
Source: PLoS One. 2023 Aug 31;18(8):e0291027. doi: 10.1371/journal.pone.0291027 (PMC10470920; doi:10.1371/journal.pone.0291027)
Supplement: S2 Table — (DOCX) [file pone.0291027.s003.docx]

S2 Table. Association between Redondovirus species in saliva and age in individuals with COVID-19 and controls who were positive for Redondovirus

Species detected Controls COVID-19

No. Mean age (SD) No. Mean age (SD)

Brisavirus + Vientovirus 15 36.8 (10.7) 9 40.8 (10.7)

Brisavirus only 6 47.2 (13.8) 10 44.7 (16.7)

Vientovirus only 8 35.9 (12.2) 8 44.6 (8.2)
